# In Vitro and In Vivo Analysis of the Mg-Ca-Zn Biodegradable Alloys

**DOI:** 10.3390/jfb15060166

**Published:** 2024-06-17

**Authors:** Bogdan Istrate, Florina-Daniela Cojocaru, Mădălina-Elena Henea, Vera Balan, Eusebiu-Viorel Șindilar, Liliana Verestiuc, Corneliu Munteanu, Carmen Solcan

**Affiliations:** 1 Mechanical Engineering, Mechatronics and Robotics Department, Mechanical Engineering Faculty, “Gheorghe Asachi” Technical University of Iasi, 700050 Iasi, Romania; bogdan.istrate@academic.tuiasi.ro; 2 Biomedical Sciences Department, Faculty of Medical Bioengineering, Grigore T. Popa University of Medicine and Pharmacy of Iasi, 9-13 Kogalniceanu Street, 700454 Iasi, Romania; balanvera@umfiasi.ro (V.B.); liliana.verestiuc@bioinginerie.ro (L.V.); 3 Surgery Unit, Clinics Department, Faculty of Veterinary Medicine, Iasi University of Life Sciences, Ion Ionescu de la Brad, 700490 Iasi, Romania; madalina.henea@yahoo.com (M.-E.H.); carmensolcan@yahoo.com (C.S.); 4 Technical Sciences Academy of Romania, 26 Dacia Blvd., 030167 Bucharest, Romania

**Keywords:** Mg-Ca-Zn alloys, in vitro analysis, biocompatibility, in vivo tests

## Abstract

The objective of this work was to analyze the in vitro and in vivo tests of a novel Mg-based biodegradable alloy—Mg-0.5%Ca—with various amounts of Zn (0.5, 1, 1.5, 2.0, and 3.0 wt.%). In terms of in vitro biocompatibility, MTT and Calcein-AM cell viability assays, performed on the MG-63 cell line through the extract method, revealed that all five alloy extracts are non-cytotoxic at an extraction ratio of 0.025 g alloy per mL of cell culture medium. In the in vivo histological analysis, Mg-0.5Ca-1.5Zn demonstrated exceptional potential for stimulating bone remodeling and showed excellent biocompatibility. It was observed that Mg-0.5Ca-0.5Zn, Mg-0.5Ca-1.5Zn, and Mg-0.5Ca-3Zn displayed good biocompatibility. Furthermore, the histological examination highlighted the differentiation of periosteal cells into chondrocytes and subsequent bone tissue replacement through endochondral ossification. This process highlighted the importance of the initial implant’s integrity and the role of the periosteum. In summary, Mg-0.5Ca-1.5Zn stands out as a promising candidate for bone regeneration and osseointegration, supported by both in vitro and in vivo findings.

## 1. Introduction

Biodegradable materials composed of magnesium (Mg) with the inclusion of zinc (Zn) and calcium (Ca) are novel biomaterials used as temporary implants [1,2].

Since each component, especially Mg, Zn, and Ca, can be found in the human body as vital minerals, the organism already possesses the necessary pathways to safely metabolize the implant [3]. Out of the three elements, Zn is the only trace element that exists in the human body, while Mg and Ca are considered bulk components. Consequently, the release of Zn has to be more rigorously regulated compared to that of Mg and Ca. By maintaining a low level of both the zinc alloy composition and its release, in combination with the degradation rate of the entire alloy, there is no need for concern [3].

The limitations of magnesium-based alloys are primarily of a mechanical nature and structural integrity in relation to the biodegradation process. In the first phase of the degradation process, hydrogen release occurs with local and temporary undesirable effects, which are later eliminated in the final phases of bone healing and regeneration.

Calcium is an essential component in the human body and has a substantial impact on the health of the bones, the circulatory system, and several cell-to-cell communication pathways [4,5]. Implanted Mg-Ca alloys may be advantageous for the tissue-healing process since Mg is essential for the absorption of Ca into bone and tissues [5,6]. Li et al. demonstrated that Mg-Ca pins exhibited no cytotoxicity towards L-929 cells and also stimulated the growth of fresh bone material in the femoral shafts of rabbits [7]. In addition, the inclusion of Ca often enhances the mechanical characteristics of Mg alloys [8]. Furthermore, a recent in vitro investigation indicated a significant enhancement in the corrosion resistance of Mg-Ca, which was linked to the presence of small amounts of calcium [9].

At the same time, Zn represents an important component for the well-functioning of the human organism, serving as a cofactor for several enzymes and playing a vital role in the immune reactions [10]. Zn has a solubility of up to 6.2 weight percent in magnesium, making it an excellent choice as an alloying component due to its very high capacity for hardening [11]. While the addition of Zn in Mg-based biomaterials enhances their tensile strength, a higher level of 1 wt.% Zn concentration might compromise the corrosion resistance of the alloy [12]. When added in low quantities, the combination of Zn and Ca was discovered to enhance corrosion resistance [13], comparable to the previously reported impact of Ca micro-alloying separately [8]. Both investigations are innovative and have been performed by applying a basic NaCl solution as a corrosive medium. Until now, it was uncertain if these consequences would manifest in the intricate setting of an in vivo scenario.

Soha et al. fabricated Mg1Zn0.6Ca, Mg2Zn0.6Ca, and Mg2.5Zn1.5Ca alloys and analyzed their microstructure, mechanical characteristics, and corrosion properties. It has been noted that there is a substantial reduction in grain size if the weight percentage of Zn and Ca in the alloy’s increases. Zn prevents the enlargement of microstructure and stimulates the formation of primary Mg grains. Adding a maximum of 2 wt.% of Zn and 0.6 wt.% of Ca results in a reduction in corrosion resistance. However, it has been demonstrated that the corrosion rate suffers a substantial rise when the amount of Ca added exceeds 6 wt.%. The electrochemical test shows that the ternary phase (Ca_2_Mg_6_Zn_3_) in Mg-2Zn-0.6Ca performs as a cathode due to its galvanic interaction with a-Mg [14].

According to Antoniac et al.’s review study [15], the binary alloy Mg-6Zn exhibited the highest corrosion resistance with an E_corr_(V) value of 1.67, an I_corr_(A) value of 122, and a corrosion rate of 2.78 mm/year. In comparison, Mg-2Zn had an E_corr_(V) value of 1.86, an I_corr_(A) value of 210, and a corrosion rate of 4.8 mm/year, while Mg-10Zn had an E_corr_(V) value of −1.74, an I_corr_(µA) value of 135, and a corrosion rate of 3.08 mm/year.

Zhang et al. [16] developed Mg-xZn-1Ca alloys by casting, where the weight percentage of Zn ranged from 1% to 6%. The researchers analyzed the mechanical and corrosion characteristics of the resulting alloys, and their research results indicated that the alloys’ yield strength, ultimate tensile strength, and ductility initially increased and then reduced as the Zn concentration increased. Increasing the zinc concentration in the alloy could result in less corrosion resistance when exposed to Hank’s solution. Out of all the alloys, the Mg-3Zn-1Ca alloy exhibits the most favorable mix of properties, having an ultimate tensile strength of 160 MPa, an elongation of 8.3%, and a corrosion rate of 2.92 mm/a. According to Li et al. [17], the tensile strength of the Mg-2Zn-xCa alloy (0 wt.% ≤ x ≤ 0.8 wt.%) first improved and subsequently declined as the Ca concentration increased.

Paul et al. [18] conducted a study on three distinct chemical compositions of Mg-Ca-Zn alloys, where the Ca content ranged from 5 to 15 wt.% and the Zn content ranged from 15 to 35 wt.%. The results of the study demonstrated that the recently produced alloys are biocompatible and able to improve cell proliferation, bringing up that the biocompatibility of the alloy with a smaller percentage of Ca and a higher amount of Zn is superior to that of the new alloys. Furthermore, the fluorescence micrographs clearly evidenced that cell morphology is uniformly distributed for all the ion-extracted media in every experimental specimen [18].

In their study, He et al. [19] examined the corrosion microstructure of the Mg-1Ca-0.5Sr-x wt.%. Zn (x = 0, 2, 4, 6) alloy after 10 days of in vitro degradation. They observed that the hydrogen evolution rate of the alloy reduced as the Zn content increased. Particularly, the alloy containing 6 wt.% Zn exhibited significant antibacterial properties [19].

Furthermore, Ding et al. [20] conducted a study on the use of Mg-Zn-Ca alloys as clips in cardiac surgery. The use of these materials in ligating the carotid artery resulted in good surgical outcomes, with no occurrence of blood leakage. No irritation to tissues or significant H_2_ gas production occurred during the deterioration of these clips. The proximity of the clip to the heart results in a higher deterioration rate compared to clips that are located farther away from the heart, due to the influence of arterial blood. Histological examination and measurement of blood biochemical indicators at several time points after clip insertion further verified the absence of tissue inflammation around the clips.

Taking into account the above-mentioned results, it can be observed that more biological studies are needed in order to fully understand the behavior of Mg-Ca-Zn biodegradable alloys and their impact on bone tissue regeneration. In this regard, the aim of this work is to evaluate in vitro and in vivo properties of Mg-0.5 wt.% Ca-x wt.% Zn (x = 0.5, 1, 1.5, 2, 3) alloys. The findings of the present investigation are expected to provide the basis for further exploration and use of these alloys.

## 2. Materials and Methods

### 2.1. Obtaining Mg-Ca-Zn Biodegradable Alloys and Previous Results

**Figure 1:**
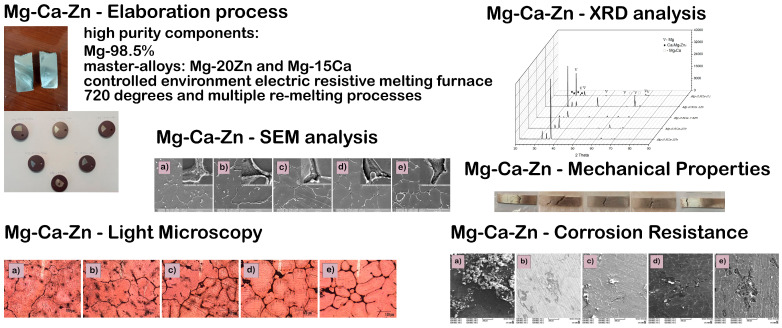
Previous results obtained on the experimental Mg-Ca-Zn alloys. Adapted from Refs. [21,22,23].

Previous investigations have conducted the production of alloys from the Mg-Ca-Zn system utilizing high-purity components, i.e., Mg-98.5%, and master-alloys, i.e., Mg-Zn (80 wt.%–20 wt.%) and Mg-Ca (85 wt.%–15 wt.%) [21,22]. The Mg-Ca-Zn alloys were produced using a controlled environment electric resistive melting furnace (SY0002-2000W-1Kg, Iasi, Romania) from the Faculty of Mechanical Engineering, Technical University Gheorghe Asachi of Iasi. The basic components have been prepared for casting and measured for each batch using an electronic balance. The experiment used cylindrical graphite crucibles, which were painted with refractory paint. The crucibles had the following dimensions: an outside diameter of 50 mm, an inner diameter of 39 mm, and a height of 100 mm. The process of melting was carried out by heating the amount of material to a temperature of 720 °C and maintaining it at this temperature for several minutes to achieve homogeneity. Inert gas (Ar) was used to purge the crucible during the melting procedures. The homogenization process included the use of a stainless steel rod to continuously mix the charge. The introduction of Zn resulted in the identification of a polyhedral structure of Mg grains with a hexagonal crystal structure, as confirmed by scanning electron microscopy and XRD patterns. The introduction of calcium resulted in the formation of spherical-shaped Zn particles (known as MgZn_2_-white compounds) and the development of the Mg_2_Ca Mg_6_Ca_2_Zn_3_ eutectic compounds at the boundary of the magnesium grains. Microstructurally, the presence of zinc leads to microstructure refinement, where the size of the grains decreases from 903.26 µm (avg. for 0.5 wt.% Zn) to 96.11 µm (avg. for 3 wt.% Zn). In Figure 1 is presented some previous results obtained by Istrate et al. on the same Mg-Ca-Zn alloy system. The corrosion rate varies between 7 and 8 mm/year, with a decreasing trend with increasing zinc content (up to 3 wt.% Zn). The corrosion products consisted of conglomerates of microcompounds with varying dimensions and shapes, mostly composed of oxides, carbonates, chlorides, salts, and phosphates [22]. The addition of zinc leads to a 3% increase in the modulus of elasticity as the concentration of Zn increases from 0.5 to 3 wt.%. Furthermore, the alloy containing 1.5 wt.% Zn has the greatest yield strength (YS) and ultimate tensile strength (UTS) values, measuring 61.13 MPa and 68.47 MPa, respectively [23].

### 2.2. Reagents for In Vitro Cytotoxicity Tests

The following reagents were used for in vitro cytotoxicity tests: Dulbecco Modified Eagles Medium Culture medium—DMEM, enriched with high glucose, pyruvate, and L-glutamate; Trypsin from pig pancreas, molecular mass 23.8 kDa; antibiotics mixture containing neomycin (1%), streptomycin (5 mg), and penicillin (5000 units); fetal serum of bovine origin—FBS; tetrazolium bromide—MTT, molecular mass 414.32 g/mol; Hanks’ Balanced saline solution—HBSS, with and respectively without calcium chloride and magnesium sulfate (HBSS Ca/Mg), with and respectively without phenol red; Calcein-AM solution in DMSO (4 mM), with a HPLC determined purity of about 90%; ethyl alcohol and dimethylsulfoxide—DMSO. Sigma-Aldrich (Darmstadt, Germany) is the manufacturer from which all the mentioned reagents were purchased.

### 2.3. Cytotoxicity Tests, In Vitro (MTT Viability Assay)

Line MG-63 (human osteosarcoma, purchased from ATCC, Rockville, MD, USA) was used to study the alloys cytotoxicity in vitro using the extract method. The alloys have the same concentration of Mg and Ca and different concentrations of Zn, and consequently were codified as follows: Zn-0.5% (Mg-0.5Ca-0.5Zn); Zn-1% (Mg-0.5Ca-1Zn); Zn-1.5% (Mg-0.5Ca-1.5Zn); Zn-2% (Mg-0.5Ca-2Zn); and Zn-3% (Mg-0.5Ca-3Zn). Table 1 presents the Mg-Ca-Zn chemical composition performed by the EDS method.

The alloys were sterilized by immersion in an alcohol solution (70%). Supplementary, the aliquots were UV irradiated for one hour on each side. Afterwards, the alloys were suspended in DMEM supplemented with 1% PSN and 10% FBS in 15 mL centrifuge tubes and kept for 24 h at 37 °C under low stirring (100 rpm). After 24 h, for supplementary sterilization, the extracts were filtered (0.22 μm), a stock solution of 0.2 mg/mL extraction ratio being obtained for each alloy, according to standard ISO 10993-12; the standard offers indications about the preparation of the samples (which are used as/part of medical devices) before their biological evaluation [24].

For MTT, the osteoblasts were cultivated at a density of 2000 cells per well in 96-well plates, while in order to study the osteoblasts morphology, the cells were cultivated at a density of 8000 cells per well in 48-well plates, in both cases being used a complete cultivation medium (DMEM + 10% BFS + 1% antibiotics mixture). The plates were placed for 24 h in an incubator with specific conditions in terms of gas concentration (5% CO_2_), temperature (37 °C), and humidity (95%). After 24 h, the medium was replaced with fresh complete DMEM medium, in the case of the control, and respectively 5 different extraction ratios expressed in g/mL (0.025, 0.05, 0.1, 0.15, and 0.2). The extraction media were put in contact with the MG-63 line and tested by MTT assay at 1, 3, and 7 days. Every 3 days the media was replaced with a fresh one (containing the mentioned extraction ratio for the test wells, respectively supplemented with DMEM for the control).

In brief, at the mentioned times, the medium from each well was aspired and replaced with the same volume of MTT solution in DMEM (0.5 mg/mL), then incubated at 37 °C for 2 h and a half. After that, the formazan crystals formed in the viable cells were solubilized with DMSO [25]. Tests were performed in triplicate, at the mentioned times being calculated the cell viability (by reporting the absorbance from test wells to the absorbance of control wells—λ = 570 nm, determined using a 96-well plate reader from Tecan (Männedorf, Switzerland). Finally, the average cell viability values ± SD (standard deviation) were graphically represented and analyzed.

### 2.4. Cell Morphology: Calcein-AM Cell Viability Assay

The test was accomplished 7 days after contact with the extracts (every 3 days the media was replaced with a fresh one, as mentioned above, for the MTT assay). For the Calcein-AM test, in a first step the medium was extracted from each well, and in order to remove the medium traces, the cells were washed using HBSS Ca/Mg without phenol red (two times). The last step was to add in each well a volume of Calcein-AM working solution (obtained by solubilizing 2 µL Calcein-AM in DMSO, 4 mM, in 1 mL HBSS Ca/Mg, without phenol red). In order to occur the reaction, the plate was reintroduced back in the incubator for 40 min. The osteoblasts morphology and density were analyzed using an inverted microscope with a fluorescence lamp from Leica (Wetzlar, Germany), 10× objective.

### 2.5. Selection of Laboratory Animals

The selection of laboratory animals was established so that as few animals as possible were used in this experimental design. Thus, two implants were used on the same rat, either at the level of both pelvic limbs or at the level of one pelvic limb and at the lumbar level, to save any animal life. In this project, 20 male Winstar rats, aged approximately 30 to 32 weeks, with a body weight of 350 ± 15 g, were randomly divided into five groups corresponding to the five concentrations taken in the study.

Pre-experimental acclimatization of the rats was ensured by housing them under constant conditions of temperature (22 ± 0.7 °C) and humidity (60 ± 10%), and the circadian cycle (light/dark) was set every 12 h.

They were kept for 5 days under laboratory conditions and were monitored daily and clinically examined for possible conditions due to transport stress or abnormal behavior. The animals were purchased from the Cantacuzino National Research and Development Institute for Microbiology and Immunology (Bucharest, Romania). At the level of the femoral region, two pieces of each alloy were implanted.

The evaluation of the soft tissue reaction around the implanted material as well as the biodegradation aspects were performed 1, 2, 4, and 8 weeks after implantation. The alloys were obtained from Mg-0.5% Ca-0.5% with variable amounts of zinc (x = 0.5, 1, 1.5, 2, 3% by weight). The alloys are in the form of cylindrical bar ingots and were cut into small-sized samples in order to be implanted in rats.

The alloys were marked as follows: alloy 1—Mg-0.5Ca-0.5Zn; alloy 2—Mg-0.5Ca-1Zn; alloy 3—Mg-0.5Ca-1.5Zn; alloy 4—Mg-0.5Ca-2Zn; and alloy 5—Mg-0.5Ca-3Zn.

The implants used had rounded corners and were parallelepipedal in shape with the following size: 10–13 mm long, 4–6 mm wide, and 1–2 mm high. Animal experiments were performed in accordance with the Guide for the Care and Use of Laboratory Animals and European animal use legislation with decision number 406/27/03/2023 issued by the Ethical Committee of the Faculty of Veterinary Medicine, “Ion Ionescu de la Brad” University of Life Sciences in Iasi, Romania.

### 2.6. Pre-Operative Preparations and Surgical Method

Rats underwent surgery under general anesthesia. Anesthesia consisted of injecting them intramuscularly with xylazine, followed by maintenance of narcolepsy with an inhaled mixture of oxygen and 1–2% isoflurane. After the induction of anesthesia, the animals were placed in sternoabdominal position on the surgical table. The area approached for implantation was trimmed and disinfected with a betadine solution.

Two anatomical regions were chosen for implantation, as one of the aims of this study was to use the implant in the human spine and long bone pathology (fractures). Two implants were used on the same rat, either in both pelvic limbs or in one pelvic limb and lumbar, to spare any animal life.

Operative access was ensured by positioning the rats on the operating table in sternoabdominal recumbency.

**Figure 2:**
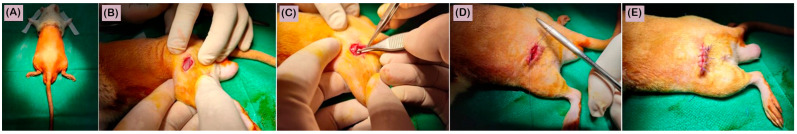
Operative steps for the surgical method: (**A**) positioning of rats on the operating table in sternoabdominal position; (**B**) skin incision; (**C**) implantation of the alloy fragment in contact with the bone tissue; (**D**) the muscle layer was sutured in a continuous thread; (**E**)the skin was sutured in separate points.

The surgical method consisted of the following operative steps (Figure 2):

The surgical method consisted of the following operative steps: incision of the cutaneous plane and fascia, muscle dilaceration, creation of a periosteal and cortical defect for the femoral diaphysis region, and a vertebral arch defect in the lumbar region of the spine. The next step was to apply the alloys, which were the same size (parallelepiped), to the defects created so that they were in direct contact with the bone tissue, periosteum, and musculature of the regions; after this step, we moved on to the next stage of tissue reconstruction, using 2/0 PGA sutures in continuous patterns to the musculature and in separate point stitches to the skin.

After surgery, the animals were allowed to move freely in their cages and were monitored daily for the visual assessment of their mobility. No side effects were observed.

## 3. Results and Discussion

### 3.1. Cytotoxicity Tests, In Vitro (MTT Viability Assay)

The MTT test is useful to study cell viability because this reagent is reduced to formazan by metabolically active cells; it passes through the cell membrane as well as the mitochondrial inner membrane of viable cells due to its positive charge as well as its lipophilic structure [26]. Although the highest tested extraction ratio (0.2 g/mL) was chosen according to the ISO 10993-12 standard, other lower ratios were also tested because, as previously mentioned by Jablonská et al. [27], a ratio of 0.2 g/mL may be inadequate for degradable materials. For instance, in the case of magnesium-based materials, it has been shown that in order to obtain relevant in vitro cytotoxicity results, the tested extraction media must be 10 times smaller [28].

**Figure 3:**
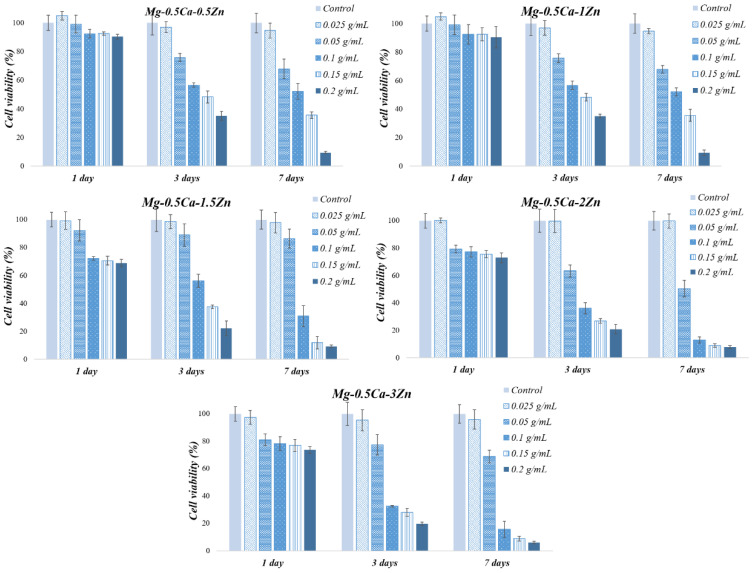
MG-63 cell viability results at 1, 3, and 7 days of contact with different alloy extraction ratios.

According to ISO 10993-5, the materials are considered non-cytotoxic if they exhibit a cell viability above 80%; weakly cytotoxic in the range of 80–60%; moderately cytotoxic at 60–40%; and strongly cytotoxic if cell viability is below 40% [28]. For all five alloy extracts (Figure 3), a decrease in cell viability can be observed during the experiment, but it is noteworthy to mention that, at a 0.025 g/mL extraction ratio, a very little decrease in cell viability was recorded over 7 days (less than 5%), indicating that all five alloys are non-cytotoxic at this quantity.

The values obtained for the 0.05 g/mL extraction ratio classify alloy 3 as non-cytotoxic, alloys 1, 2, and 5 as weakly cytotoxic, and alloy 4 as a material with moderate cytotoxicity, whereas the results obtained for the extracts with a 0.1 g/mL extraction ratio place alloy 1 in materials with moderate cytotoxicity, alloy 2, on the border between moderate cytotoxicity and strong cytotoxicity, and alloys 3, 4, and 5 in materials with strong cytotoxicity.

This cytotoxic effect observed for the maximum extraction ratio tested (the undiluted one, suggested by standard ISO 10993, parts 5 and 12) was also reported previously by Li et al. [29] for degradable metallic alloys (principally based on Zn). This common observation can be linked with the fact that the mentioned standard was mainly introduced for non-absorbable biomaterials, which is not the case with Mg-Zn-Ca alloys. Moreover, since the extract method was chosen, it is clear that cytotoxicity is strongly influenced by the degradation products and also other alloy properties, such as corrosion resistance [29]. More precisely, the Mg alloys corrosion reaction is based on the consumption of H^+^ ions, resulting OH^-^ ions, followed by an increase of medium pH > 8.4. This value is inappropriate for osteoblasts, which proliferate at a pH between 8 and 8.4 [30].

However, it is important to note that these results need to be correlated also with in vivo tests, since it is known that the second are more complex and express more carefully the body response to implanted materials.

### 3.2. Cell Morphology: Calcein-AM Cell Viability Assay

**Figure 4:**
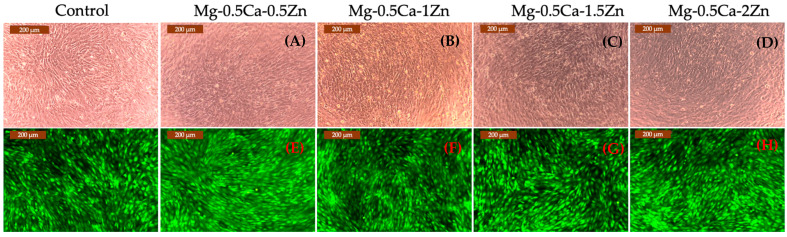
MG-63 cells morphology in wells, incubated with extracts (0.025 g/mL): without staining, the upper side; and with Calcein-AM staining, the lower side: Mg-0.5Ca-0.5Zn (**A**,**E**); Mg-0.5Ca-1Zn (**B**,**F**); Mg-0.5Ca-1.5Zn (**C**,**G**); Mg-0.5Ca-2Zn (**D**,**H**) (scale bar 200 μm).

After 7 days of contact, the cell morphology was analyzed, without staining, in a first step, and the results are shown in Figure 4A–D and with Calcein-AM staining in a second step (Figure 4E–H). From the images, it can be observed that a comparable density of cells in the wells incubated with 0.025 g/mL extraction ratio extracts to that of control wells and no significant modification of shape cells. The cell morphology and density are correlated with the MTT data, since for all alloys, there were registered viability values above 90% at 0.025 g/mL extraction ratio.

As a conclusion for the two cytotoxicity assays, the obtained data indicated that after 7 days, for the smaller extraction ratio tested, all the materials are free of cytotoxicity, suggesting that the alloy degradation products have no harmful effects on osteoblasts. On the other hand, for the next tested extraction ratio, 0.05 mg/mL was observed to have the best behavior for alloy 3, containing 1.5 wt.% Zn. These data can be correlated with those reported before by the research group [23] and briefly presented in Section 2.1, where it was mentioned that this alloy has superior mechanical behavior in comparison with the other 4.

### 3.3. Histologic Analysis and Characterization

Samples were cut into small sections, fixed in a 10% formalin solution for 2 days, demineralized with 10% vol. trichloroacetic acid, dehydrated with alcohol in ascending concentrations, and embedded in paraffin. For histological studies, 5 µm thick sections were obtained from the area near the implant and prepared for staining with hematoxylin and eosin (H&E). The stained sections were examined under a light microscope.

The healing process/osteocyte response of the periosteum and bone tissue adjacent to the implant and the performance of the implant material were assessed by histological examination. Histological analysis focused on highlighting the morphology of the periosteum and peri-implant bone area, starting with the healing stages at the first week, second week, fourth week, and eighth week after implant placement. Therefore, aspects of osseointegration such as cell proliferation, differentiation and classification of cell types, healing processes, and bone remodeling were considered. The evaluation was carried out in accordance with in vitro studies.

**Figure 5:**
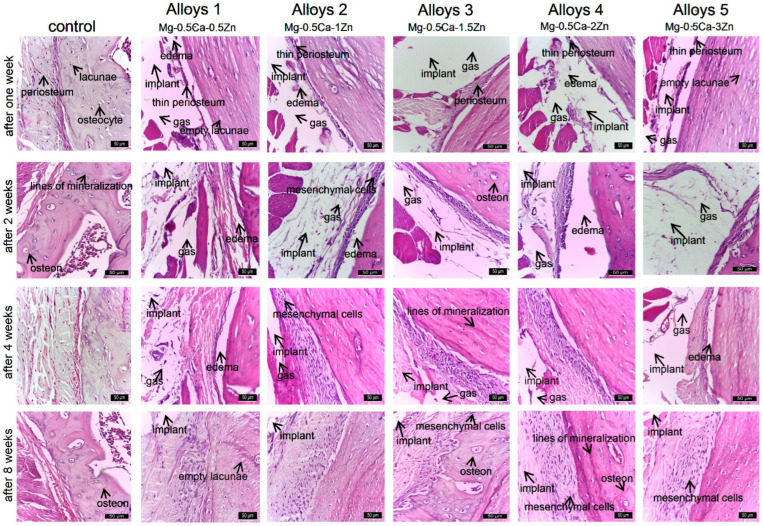
Histological aspects of periosteum and peri-implant bone tissue at 1, 2, 4, and 8 weeks in rats implanted with alloy 1–5. H&E stain ×400.

Bones from the control group showed a characteristic compact bone tissue structure. At the periphery, a periosteum consisting of an outer layer with vascular and intensely innervated structures was observed. The inner part of the periosteum consists of stem cells, connective fibers, and extracellular matrix. After mitotic division, stem cells differentiate into two cell types, i.e., some resident cells that remain as a reserve and others that differentiate into osteoblasts. The latter are involved in the synthesis of bone matrix and collagen fibers in the peripheral region of compact bone. After synthesis, osteoblasts close the gaps and become osteocytes, which play an important role in bone metabolism and mineralization. Compact bone tissue consists of osteons with a central Havers canal and bone lamellae arranged concentrically around this canal. Cementation lines appear between the osteons (Figure 5, column 1).

In the implant alloy 1, at first and second weeks after implant placement, the peripheral area is characterized by a thin periosteum, detached from the bone by edema (Figure 5, coumn 2). The lamellar bone tissue at the periphery frequently shows hollow gaps without osteocytes. After four weeks, the peri-implant area consists of an outer, vascularized fibrous layer with numerous activated mesenchymal cells. In the bone laminae, elongated osteocytes were observed in the lacunae, rarely thin and incomplete mineralization lines; after eight weeks, the periosteum was repaired, and the outer layer was composed of fibers and connective cells, extracellular matrix, and blood vessels. The diameter of the periosteal layer was larger than after four weeks. The interior of the periosteum showed an area with numerous activated mesenchymal cells, an outer fibrous layer, blood vessels, and an extracellular matrix. Elongated osteocytes and thin and incomplete mineralization lines were observed in the lacunae. After 8 weeks in the inner periosteum, differentiated mesenchymal and osteogenic cells were observed. The bone lamellae were organized circularly, forming small osteons with 1–2 bone lines arranged around the Havers canal. In the bony region near the repaired periosteum, mineralization lines (cementum lines) and osteocytes were evidentiated within the lacunae. Lamellar bone formation and parallel mineralization lines separating the osteons from the interstitium were observed. The lumen contained numerous active cells that synthesize the extracellular matrix of bone tissue. Blood vessels were present inside the bone.

In implant alloy 2, the first and second weeks after implant placement, the periphery was characterized by a thin periosteum that has detached from the bone due to reduced edema (Figure 5, column 3). The peripheral bone tissue was lamellar, without osteocytes and with many empty lacunae; after the fourth week, the periosteum was thickened on the basis of mesenchymal cells, and the newly formed bone tissue showed an active remodeling process, with osteocytes arising parallel to the fissure, with a clear core and thin, sparse, and incomplete mineralization lines. Eight weeks after implantation, the periosteum was slightly thicker than in the previous two alloys, and some bone lamellae parallel with the periosteum; obvious cementum lines were also observed. The bone lamellae were arranged in a circular pattern constituting the primary osteon, which consisted of a small number of bone lamellae. The periosteal fibrous tissue was temporarily replaced by the surface lamellar bone.

One and two weeks after implantation of alloy 3, a thin periosteum was evident, detached from the bone due to peripheral edema (Figure 5, column 4). Mesenchymal cells in the inner layer of the periosteum continued to differentiate into osteogenic cells. The outer periosteum consisted of a thin fibrous layer. Mineralization lines appeared in the newly formed peripheral bone tissue. Bone laminae in this area showed gaps in which osteocytes were located. The number of lacunae occupied by osteocytes was higher than in other experimental groups. Four weeks after implantation, a continuous fibrous periosteum was observed around the implant, newly formed trabecular bone was remodeled, and osteocytes in the lacunae were large, having obvious nuclei, and being metabolically active. Mineralization lines were more numerous and longer. After 8 weeks, the periosteum was attached to bone tissue and had a normal appearance. Bone lamellae were attached parallel to the periosteum and were distinguished by obvious cementum lines. The bone lamellae were arranged circularly, with a small number of lamellae making up the primary bone. The bone matrix was partially mineralized.

In the implant alloy 4, after the first and second weeks of the experiment, the periphery showed thin periosteum edema (Figure 5, column 5) and frequent gaps without osteocytes in the peripheral lamellar bone tissue. At the fourth week, the lacunae were occupied by osteocytes, and thin and incomplete mineralization lines became visible. The reduced rate of deposition of bone matrix and collagen fibers resulted in the appearance of interlamellar gaps. After 8 weeks, the periosteum was slightly thicker than before, and the bone lamellae were arranged parallel or concentric to the periosteum, with a few lamellae encompassing the primary osteons, their number being reduced.

In the implant alloy 5, the periphery was characterized by a thin periosteum after the first week and second week of the experiment, with edema formed between the periosteum bone and the musculature (Figure 5, column 6). In the peripherally arranged lamellar bone tissue, hollow lacunae devoid of osteocytes were frequently observed. After four weeks, the periosteum was partially recovered, and osteocytes were sparse, reduced in volume, and mineralization lines were incomplete. At eight weeks, periosteal thickness was somewhat greater than at four weeks. The periosteum consisted of an outer fibrous zone and inner activated stem cells, with some bone lamellae parallel to the periosteum. Mineralized and non-mineralized bone and mineralized lines alternated in this area.

In the five implants (Figure 5), all the changes were similar, excepting the implant alloy 3, where the periosteal cells were larger and more numerous than in the other groups; the changes after four and eight weeks were favorable, and finally bone mineralization was observed. In this group, a newly formed small periosteum appeared; histological examination at first week showed good compliance of all implants used, inflammatory reactions, reduced edema, and little gas between the thin periosteum and bone tissue; at two weeks, a thin layer of mesenchymal stem cells was observed, which proliferated and formed a thicker layer at four weeks after implant placement; by four weeks, small spaces probably with gas were observed; at eight weeks, the periosteal cell layer was thicker than in the control group. The alloy 3 implant has the best ability to induce bone remodeling compared to other implants. The gradual recovery of the periosteum, vascularization of the peri-implant sheath, bone hardening, and the formation of longer, more complete, and parallel demineralization lines suggest that alloy 3 has very good biocompatibility, followed by alloys 1, 2, 4, and 5.

**Figure 6:**
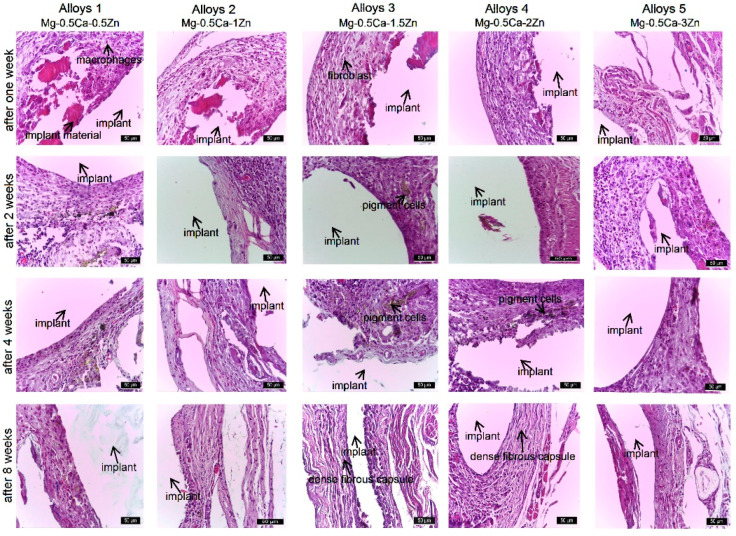
Reactivity of peri-implant connective tissue capsulae during the experiment. H&E stain ×400.

The periosteum was partially detached, and the biomaterial was attached to the external bone surface. After four weeks, the capsule was identified but was displaced 150–200 µm from the periosteum. The space around the conjunctival capsule to the periosteum was occupied by a reduced gas accumulation in the connective tissue, which was significantly reduced at the eighth week. At eight weeks, the conjunctival capsule was thickened, consisting of fibroblasts, collagen fibers, and newly formed vessels. Some cells of the conjunctival capsule showed pigments as a result of biomaterial uptake. A higher frequency of these cells was observed in the alloy 3 group (Figure 6).

Histological staging of material precursors at the implant site at the first, second, fourth, and eighth weeks post-implantation was as follows: At the first week, there were reduced connective tissues, a partially resorbed area of implant material surrounded by peripheral fibrotic reactions, and a moderate inflammatory reaction including macrophages, fibroblasts, and collagen fibers. After the second week, there were low absorption of implant material and gas release, moderate inflammatory reaction around implant material, presence of collagen fibers (young connective tissue), and newly formed capillaries. In weeks four and eight after implant placement, significant resorption of the implanted material was accompanied by a reduction in gas release, visualized as hollow spaces surrounded by periosteum and bone and connective tissue around the implant. Gas release was proportional to the absorption rate of the alloy; after eight weeks, the connective tissue at the implant site was well organized, replacing the absorbed implant material. In vivo studies of magnesium-based alloys with different Zn concentrations showed very good histocompatibility and tissue resorption after eight weeks. Sparse, short, and discontinuous interlamellar areas two weeks after implant placement reflect gradual recovery of the periosteum, discontinuous matrix deposition associated with desynchronized osteoblast synthesis [31], and reduced deposition rates [32]. In lamellar bone, four weeks after implant placement, mineralization lines were observed as dark stripes between bone lamellae, which are said to represent areas of low matrix density [33] or reduced collagen fiber organization [34]. These changes highlight that implants caused destruction of periosteum and periosteal bone within the first four weeks. Four to eight weeks after implant placement, the bone tissue was better organized and characterized by evenly oriented mineralization lines over long distances.

The osteocytes were highly organized and had a more elongated ellipsoidal shape with parallel axes arranged in a matrix, as described in the literature [35]. Osteocytes were involved in bone mineralization in all groups, but especially in alloy 3.

The process of bone remodeling ensures continuous bone regeneration throughout life by synchronizing and coordinating bone resorption and osteosynthesis activities. In the study group, there was active histological enhancement of peri-implant bone tissue, suggesting good osseointegration. This is justified by the identified areas of physiological bone and periosteal healing, with the formation of immature bone tissue and its transformation into mature, lamellar bone tissue. The rate of bone turnover is very rapid in young organisms, about 200 times faster than in adults. Clinical studies suggest that when implants are placed in bone tissue, the periosteum responds by cell proliferation, which promotes bone formation within a few days of placement, thus ensuring initial implant integrity [36].

The corrosion rate of magnesium is excessively high. Magnesium corrosion is associated with hydrogen release, which can complicate the healing process. Therefore, the corrosion rate of Mg is reduced by alloying [37]. The mechanical properties of zinc are similar to those of magnesium, but the corrosion rate is significantly lower. In addition, zinc corrosion does not produce hydrogen, which is beneficial for wound healing [38]. A good healing capacity and reduced gas release were also observed in prefixation experiments. The frequency of inflammatory cells also increased in the first and second week groups. The number of fibroblasts and periosteal thickness were almost double in the experimental group compared to the control one. Inflammatory cells were no longer present, and the periosteum was more compact. The interface was smooth and almost completely adherent, and edema was eliminated.

## 4. Conclusions

This work evaluated in vitro and in vivo behavior of novel Mg-based biodegradable alloys—Mg-0.5%Ca—with various amounts of Zn (0.5, 1, 1.5, 2.0, and 3.0 wt.%). In terms of in vitro biocompatibility evaluation, performed on the MG-63 cell line through the extract method and expressed as MTT and Calcein-AM cell viability results, it was revealed that all five alloy extracts are non-cytotoxic at a 0.025 g/mL extraction ratio. The five alloys showed good biocompatibility and did not induce inflammatory, rejection, or other pathological changes. Of all, alloy 3 showed the best compatibility from the beginning. It induced remodeling of the periosteum and the appearance of osteons with a mineralized bone matrix at 8 weeks post-implantation.

Magnesium alloys are considered to be suitable for application in medicine as biodegradable implant materials, such as bone fracture fixation devices. Mg-based alloys have as their main advantage the ability to gradually degrade without affecting the patient and also to be fully absorbed by the animal body.

Finally, the Mg-0.5Ca-1.5Zn alloy proved to be a promising biodegradable implant for orthopedic applications in terms of bioactivity and compatibility with human bone. The rate of material degradation should be optimized according to the type of fracture and the estimated healing time.

## Figures and Tables

**Table 1:** Chemical composition results by Bruker EDS method.

Alloy	Mg [wt.%]	Ca [wt.%]	Zn [wt.%]
Mg-0.5Ca-0.5Zn	99.05	0.43	0.52
Mg-0.5Ca-1Zn	98.40	0.56	1.04
Mg-0.5Ca-1.5Zn	98.10	0.51	1.39
Mg-0.5Ca-1Zn	97.64	0.62	1.74
Mg-0.5Ca-3Zn	96.22	0.63	3.15

EDS analysis performed in 5 separate areas.

## Data Availability

The original contributions presented in the study are included in the article, further inquiries can be directed to the corresponding author.

## References

[1] Sommer N.G., Hirzberger D., Paar L., Berger L., Ćwieka H., Schwarze U.Y., Herber V., Okutan B., Bodey A.J., Willumeit-Römer R. (2022). Implant degradation of low-alloyed Mg–Zn–Ca in osteoporotic, old and juvenile rats. Acta Biomater..

[2] Rout P.K., Roy S., Rathore D. (2023). Recent advances in the development of Mg-Ca-Zn alloys as biodegradable orthopedic implants. Mater. Today Proc..

[3] Zhang J., Li H., Wang W., Huang H., Pei J., Qu H., Yuan G., Li Y. (2018). The degradation and transport mechanism of a Mg-Nd-Zn-Zr stent in rabbit common carotid artery: A 20-month study. Acta Biomater..

[4] Sahu M.R., Kumar T.S.S., Chakkingal U. (2022). A review on recent advancements in biodegradable Mg-Ca alloys. J. Magnes. Alloys.

[5] Renkema K.Y., Alexander R.T., Bindels R.J., Hoenderop J.G. (2008). Calcium and phosphate homeostasis: Concerted interplay of new regulators. Ann. Med..

[6] Li N., Zheng Y. (2013). Novel magnesium alloys developed for biomedical application: A review. J. Mater. Sci. Technol..

[7] Li Z., Gu X., Lou S., Zheng Y. (2008). The development of binary Mg–Ca alloys for use as biodegradable materials within bone. Biomaterials.

[8] Basu I., Chen M., Wheeler J., Schaublin R.E., Loffler J.F. (2022). Segregation-driven exceptional twin-boundary strengthening in lean Mg–Zn–Ca alloys. Acta Mater..

[9] Deng M., Wang L., Hoche D., Lamaka S.V., Wang C., Snihirova D., Jin Y., Zhang Y., Zheludkevich M.L. (2021). Approaching “stainless magnesium” by Ca micro-alloying. Mater. Horiz..

[10] Hojyo S., Fukada T. (2016). Roles of Zinc Signaling in the Immune System. J. Immunol. Res..

[11] Satya Prasad S.V., Prasad S.B., Verma K., Mishra R.K., Kumar V., Singh S. (2022). The role and significance of Magnesium in modern day research—A review. J. Magnes. Alloys.

[12] Cihova M., Martinelli E., Schmutz P., Myrissa A., Schaublin R., Weinberg A.M., Uggowitzer P.J., Loffler J.F. (2019). The role of zinc in the biocorrosion behavior of resorbable Mg–Zn–Ca alloys. Acta Biomater..

[13] Skolakova A., Lovasi T., Pinc J., Kacenka Z., Rieszova L., Zofkova Z. (2020). The effect of zinc and calcium addition on magnesium alloy. Manuf. Technol..

[14] Abdel-Gawad S.A., Shoeib M.A. (2019). Corrosion studies and microstructure of Mg-Zn-Ca alloys for biomedical applications. Surf. Interfaces.

[15] Antoniac I., Miculescu M., Mănescu (Păltânea) V., Stere A., Quan P.H., Păltânea G., Robu A., Earar K. (2022). Magnesium-Based Alloys Used in Orthopedic Surgery. Materials.

[16] Zhang B.P., Hou Y.L., Wang X.D., Wang Y., Geng L. (2011). Mechanical properties, degradation performance and cytotoxicity of Mg–Zn–Ca biomedical alloys with different compositions. Mater. Sci. Eng. C.

[17] Li H.X., Qin S.K., Yang C.L., Ma Y.Z., Wang J., Liu Y.J., Zhang J.S. (2018). Influence of Ca addition on microstructure, mechanical properties and corrosion behavior of Mg-2Zn alloy. China Foundry.

[18] Paul S., Ramasamy P., Das M., Mandal D., Renk O., Calin M., Eckert J., Bera S. (2020). New Mg-Ca-Zn amorphous alloys: Biocompatibility, wettability and mechanical properties. Materialia.

[19] He G., Wu Y., Zhang Y., Zhu Y., Liu Y., Li N., Zheng Y. (2015). Addition of Zn to the ternaryMg–Ca–Sr alloys significantly improves their antibacterial properties. J. Mater. Chem. B..

[20] Ding P., Liu Y., He X., Liu D., Chen M. (2019). In vitro and in vivo biocompatibility of Mg–Zn–Ca alloy operative clip. Bioact. Mater..

[21] Lupescu S., Istrate B., Munteanu C., Minciuna M.G., Focsaneanu S., Earar K. (2017). Characterization of Some Master Mg-X System (Ca, Mn, Zr, Y) Alloys Used in Medical Applications. Rev. Chim..

[22] Istrate B., Munteanu C., Bălțatu M.-S., Cimpoeșu R., Ioanid N. (2023). Microstructural and Electrochemical Influence of Zn in MgCaZn Biodegradable Alloys. Materials.

[23] Istrate B., Benchea M., Goanta V., Munteanu C., Baltatu M.S. (2023). Study of the tribological and mechanical properties of some biodegradable Mg-Ca-Zn alloys. Int. J. Mod. Manuf. Technol..

[24] (2009). Biological Evaluation of Medical Devices—Part 12: Sample Preparation and Reference Materials.

[25] Kumar P., Nagarajan A., Uchil P.D. (2018). Analysis of cell viability by the MTT assay. Cold Spring Harb. Protoc..

[26] Ghasemi M., Turnbull T., Sebastian S., Kempson I. (2021). The MTT Assay: Utility, Limitations, Pitfalls, and Interpretation in Bulk and Single-Cell Analysis. Int. J. Mol. Sci..

[27] Jablonská E., Kubásek J., Vojtěch D. (2021). Test conditions can significantly affect the results of in vitro cytotoxicity testing of degradable metallic biomaterials. Sci. Rep..

[28] (2009). Biological Evaluation of Medical Devices. Part 5: Tests for In Vitro Cytotoxicity.

[29] Li P., Schille C., Schweizer E., Kimmerle-Müller E., Rupp F., Heiss A., Legner C., Klotz U.E., Geis-Gerstorfer J., Scheideler L. (2019). Selection of extraction medium influences cytotoxicity of zinc and its alloys. Acta Biomater..

[30] Cho D.H., Avey T., Nam K.H., Dean D., Luo A.A. (2022). In vitro and in vivo assessment of squeeze-cast Mg-Zn-Ca-Mn alloys for biomedical applications. Acta Biomater..

[31] Stein K., Prondvai E. (2013). Rethinking the nature of fibrolamellar bone: An integrative biological revision of sauropod pelxiform bone formation. Biol. Rev. Camb. Philos. Soc..

[32] Prondvai E., Stein K.H.W., De Ricqlès A., Cubo J. (2014). Development-based revision of bone tissue classification: The importance of semantics for science. Biol. J. Linn. Soc..

[33] Marotti G., Ferretti M., Palumbo C. (2013). The problem of bone lamellation: An attempt to explain different proposed models. J. Morphol..

[34] Bromage T., Lacruz R.S., Hogg R., Goldman H.M., McFarlin S.C., Warshaw J., Dirks W., Perez-Ochoa A., Smolyar I., Enlow D.H. (2009). Lamellar bone is an incremental tissue reconciling enamel rhythms, body size, and organismal life history. Calcif. Tissue Int..

[35] Kerschnitzki M., Wagermaier W., Roschger P., Seto J., Shahar R., Duda G.N., Mundlos S., Fratzl P. (2011). The organization of the osteocyte network mirrors the extracellular matrix orientation in bone. J. Struct. Biol..

[36] Pazzaglia U.E. (1996). Periosteal and endosteal reaction to reaming and nailing: The possible role of revascularization on the endosteal anchorage of cementless stems. Biomaterials.

[37] Dvorský D., Kubásek J., Čapek J., Pinc J., Vojtěch D. (2019). Characterization of Zn-1.5 Mg and Zn-1.5 Mg-0.5 Ca Alloys Considered for Biomedical Application. Key Eng. Mater..

[38] Jin H., Zhao S., Guillory R., Bowen P.K., Yin Z., Griebel A., Schaffer J., Earley E.J., Goldman J., Drelich J.W. (2018). Novel high-strength, low-alloys Zn-Mg (<0.1wt% Mg) and their arterial biodegradation. Mater. Sci. Eng. C.

